# Dietary Linolenic Acid Increases Sensitizing and Eliciting Capacities of Cow’s Milk Whey Proteins in BALB/c Mice

**DOI:** 10.3390/nu14040822

**Published:** 2022-02-16

**Authors:** Xuanyi Meng, Yong Wu, Xuefang Wen, Jinyan Gao, Yanhai Xie, Xiaoli Zhao, Jin Yuan, Hao Yang, Zheling Zeng, Xin Li, Hongbing Chen

**Affiliations:** 1 State Key Laboratory of Food Science and Technology, Nanchang University, Nanchang 330047, China; mengxuanyilucky@163.com (X.M.); ericyo918@hotmail.com (Y.W.); gaojy2013@ncu.edu.cn (J.G.); xieyh818@sina.com (Y.X.); x.zhao1@uu.nl (X.Z.); yuan2jin@126.com (J.Y.); 13217917823@163.com (H.Y.); zlzengjx@163.com (Z.Z.); zhizilixin@ncu.edu.cn (X.L.); 2 Sino-German Joint Research Institute, Nanchang University, Nanchang 330047, China; 3 Instiute of Applied Chemistry, Jiangxi Academy of Sciences, Nanchang 330096, China; wxf198508@163.com; 4 School of Food Science & Technology, Nanchang University, Nanchang 330031, China; 5 Jiangxi Province Key Laboratory of Edible and Medicinal Resources Exploitation, Nanchang University, Nanchang 330031, China

**Keywords:** polyunsaturated fatty acids, linolenic acid, whey proteins, components interaction, allergic reaction

## Abstract

α-Lactalbumin (BLA) and β-lactoglobulin (BLG) are the major whey proteins causing allergic reactions. Polyunsaturated fatty acids (PUFAs) stand among the extrinsic factors of the food matrix that can bind BLA and BLG and change their bioactivities, but their contribution to change the allergenic properties of these proteins has not been investigated. Here, we aimed to determine how PUFAs influence BLA and BLG to sensitize and trigger allergic responses in BALB/c mice. First, tricine–SDS–PAGE and spectroscopic assays identified that α-linolenic acid (ALA, as a proof-of-concept model) can induce BLA and BLG to form cross-linked complexes and substantially modify their conformation. Then, BALB/c mice (*n* = 10/group) were orally sensitized and challenged with BLA and BLG or ALA-interacted BLA and BLG, respectively. Allergic reactions upon oral challenge were determined by measuring clinical allergic signs, specific antibodies, levels of type-1/2 cytokines, the status of mast cell activation, and percentage of cell populations (B and T cells) in different tissues (PP, MLN, and spleen). Overall, systemic allergic reaction was promoted in mice gavage with ALA-interacted BLA and BLG by disrupting the Th1/Th2 balance toward a Th2 immune response with the decreased number of Tregs. Enhanced induction of Th2-related cytokines, as well as serum-specific antibodies and mast cell activation, was also observed. In this study, we validated that ALA in the food matrix promoted both the sensitization and elicitation of allergic reactions in BALB/c mice.

## 1. Introduction

Food allergy is a global health issue, with increasing prevalence, and is currently estimated to affect 5% of adults and 6–8% of children [1,2,3]. Considering the absence of a cure, the standard food allergy management is strictly dietary avoidance, which is impossible to maintain because of the recurrence of accidental exposures in individuals with food allergies [4,5]. Considering that food-allergic reactions occur following allergic proteins ingestion or inhalation, modifying the allergic protein’s structure to change its immunogenic and sensitizing capacities is an important research strategy [6]. Therefore, the association between different food processing methods and modifying the allergenic properties of allergenic proteins have extensively been studied [7,8,9]. However, most studies related to food allergens have used the pure forms of allergens (intrinsic factors), which may not be physiologically relevant because allergenic proteins sensitization is a multifactorial process [10]. Specifically, this sensitization process depends not only on the allergen per se but also on other molecular compounds (extrinsic factors) within concomitantly ingested or inhaled allergen sources, such as lipids [11]. Lipids can act as adjuvants that activate the innate immunity or skew allergen-specific immune response toward Th2 by dampening allergen degradation in the gastrointestinal tract, which facilitates the uptake of allergens by the intestinal epithelial cells [11,12,13]. Therefore, the further effect of lipids on the sensitizing and eliciting capacity of food allergens in a mouse model needs to be elucidated.

Cow’s milk (CM) is among the most potent allergenic foods of animal origin and is frequently used in the food industry to prepare various food products, such as yogurt and cheese, that contain high amounts of fat [14]. Epidemiological reports indicated an increase in the prevalence of CM allergy (CMA), which affects approximately 2.5% of infants and approximately 0.4–0.9% of the whole population [15]. Although patients with CMA are polysensitized to several CM proteins, α-lactalbumin (BLA) and β-lactoglobulin (BLG) are considered the dominant milk allergens [16]. Fatty acids (FAs), especially C18 polyunsaturated FAs (PUFAs) constitute 30–40% of the total milk FAs [17], which are released in substantial amounts from dietary fat in the upper gastrointestinal tract and can interact with BLA and BLG to form protein–ligand complexes through electrostatic, hydrophobic cavities, or hydrophobic interactions [18,19]. A growing literature demonstrated that FA–BLA/BLG complexes have special biological properties that can induce apoptosis in tumor and immature cells but not in healthy differentiated cells [18,20,21,22]. Additionally, previous reports have elucidated that PUFAs (especially ω-3 PUFAs) could prevent food allergy, mainly because they are able to target almost every cell type within the sensitization and effector phases [23]. For example, Elsen et al. showed that the ω-3 PUFAs diet could generate functional Treg and suppress Th2 cell frequency in the MLN and/or the spleen in whey-sensitized mice [24]. However, for PUFAs, the importance of the capacity of the formed complexes to modify the conformation of BLA/BLG and affect their allergenic capacity remains unclear.

Accordingly, we hypothesized that PUFAs in diet can modify the allergenic capacity of BLA and BLG. In this study, we chose α-linolenic acid (ALA, ω-3 PUFAs, 8–15% of C18 PUFAs [17]) as a proof-of-concept model because humans cannot synthesize ALA, and ALA is an essential dietary component to investigate whether and how ALA-interacted BLA/BLG (ALA–BLA/ALA–BLG) complexes affect oral sensitization in naïve mice. We also investigated immune responses to oral antigen challenges in sensitized mice. Our results here demonstrated that, in addition to the interaction with CM proteins, PUFAs promote the sensitizing and eliciting capacity of these proteins in mice.

## 2. Materials and Methods

### 2.1. ALA–BLA/ALA–BLG Complex Preparation

ALA–BLA/ALA–BLG complexes were prepared using our previous protocols, with some modifications [25]. First, bovine BLA/BLG solution was produced in two concentrations. A total of 4 g lyophilized BLA (purity ≥ 85%, Sigma-Aldrich, St. Louis, MO, USA) or BLG (purity ≥ 90%, Sigma-Aldrich, St. Louis, MO, USA) were dissolved in 100 mL of PBS (pH of 7.4) to obtain 40 mg/mL BLA/BLG solution, and 3.5 g lyophilized BLA/BLG was dissolved in 35 mL of PBS (pH of 7.4) to make 100 mg/mL BLA/BLG solution. To prepare the ALA–BLA/ALA–BLG complexes, we directly added 50 (40 mg/mL) and 17.5 mL (100 mg/mL) of BLA/BLG solutions to ALA (α-linolenic acid, purity ≥ 98%, Cayman Chemical item no. 90210, CAS no. 463-40-1) at a 50 molar equivalent ratio (i.e., 50 mol of FA per mol of protein). Then, the mixtures were vortexed for 30 s and then incubated at 60 °C in a water bath for 30 min. Excess ALA in the complexes was carefully removed by centrifugation at 9000× *g* at 4 °C for 20 min, followed by dialysis against milli-Q water (Millipore, MA, USA) for 2 days at 4 °C. The water was changed two times a day, and the dialyzed retentate was stored at −20 °C until use. The binding ratio was determined with the Free Fatty Acid Quantification Kit (Biovison, Mountain View, CA, USA), according to the manufacturer’s instructions, and the molar ratios of both ALA/BLA and ALA/BLG were 9–10 (Figure S1), which were consistent with previous observations [25]. The same volume of untreated BLA/BLG (no ALA and without heating) solution was also stored at −20 °C until use. BLA/BLG and ALA–BLA/ALA–BLG complex solutions were vortexed thoroughly before use, to ensure that all solutions were homogenous.

### 2.2. ALA–BLA/ALA–BLG Complex Characterization

To determine the status of protein composition, we analyzed ALA–BLA/ALA–BLG complex samples through nonreducing tricine–sodium dodecyl sulfate–polyacrylamide gel electrophoresis (tricine–SDS–PAGE) [26], by using a discontinuous system containing 4%, 10%, and 16.5% acrylamide with a constant voltage in the range of 30–100 V. The gels were immersed in a fixative solution containing ethanol, acetic acid, and water (4:1:5 *v*/*v*/*v*) for 30 min, followed by staining with Coomassie Blue solution (0.5 g Coomassie Blue, 40% methanol, 10% acetic acid, and 50% distilled water) and distained with a solution containing 5% methanol and 7.5% acetic acid. Then, the gels were immersed in distilled water until the background became clear, followed by taking images by using a ChemiDoc imager (Bio-Rad, Hercules, CA, USA).

To determine the secondary and tertiary structures of the ALA–BLA/ALA–BLG complex, we used circular dichroism (CD) spectroscopy, ultraviolet (UV) absorption spectroscopy, and fluorescence spectroscopy in this study. CD measurement was recorded in a wavelength range of 190–250 nm, with a resolution of 0.2 nm, a bandwidth of 1.0 nm, a scan speed of 60 nm/min, and a path length of 0.1 cm. The samples were dissolved in PBS (pH of 7.4) to a final concentration of 0.2 mg/mL. The α-helix, β-sheet, and random coil percentages were calculated using the CD Pro software (JASCO, Tokyo, Japan) provided along with the instruments (JASCO J-715 spectropolarimeter, Japan Spectroscopy Co., Tokyo, Japan.).

For UV absorption spectroscopy analysis, protein samples were prepared in 10 mM PBS (pH of 7.4) at a concentration of 0.2 mg/mL. The spectrum was recorded in the range of 230–350 nm by using a UV spectrophotometer (TU-1901, Purkinje General, Beijing, China). Background corrections were made with PBS without protein in all cases.

For fluorescence spectroscopy analysis, the protein-bound 8-anilinonaphthalene-1-sulfonic acid (ANS, Sigma-Aldrich) fluorescence was used to monitor the surface hydrophobic character of the protein binding. Optimized ANS concentration (8 mM, 50 μL) was added to each protein sample (0.2 mg/mL, 5 mL) prepared in PBS (pH of 7.4). In all cases, each mixture sample was vortexed and incubated for 50 min at room temperature in the dark and then recorded using a fluorescence spectrophotometer (F-4500, Hitachi Limited, Tokyo, Japan). BLA/BLG and ALA–BLA/ALA–BLG complex bound ANS fluorescence emission spectra were measured from 400 nm to 650 nm, at an excitation wavelength of 350 nm.

### 2.3. Animals and Experimental Design

The 3–4 weeks old female-specific pathogen-free BALB/c mice with body weight of 18.0 ± 2.0 g were purchased from Hunan Slac Jingda Laboratory Animal Co. (Changsha, China, Certificate number: SCXK (Xiang) 2016-0002). This study was carried out in strict accordance with the recommendations in the National Guide for the Care and Use of Laboratory Animals of China. All experimental procedures were approved by the Animal Care Review Committee, Nanchang University. These mice were housed in an animal room with a controlled temperature of 23 ± 1 °C, relative humidity of 50 ± 5%, and 12/12 h of light–dark cycle with ad libitum access to water and commercially available rodent food (milk-protein-free). The mice were housed with 10 mice/cage in polypropylene cages (32 cm × 20 cm × 14 cm) and provided ad libitum access to a commercially available rodent diet and water. Water was made available continuously through automatic ports, and the diet did not contain any milk allergens that could influence the outputs of the experiments. Moreover, the bedding (wood shaving) of mice was replaced every two days. The mice were acclimatized to the laboratory condition 1 week before the start of the animal experiment.

**Figure 1:**
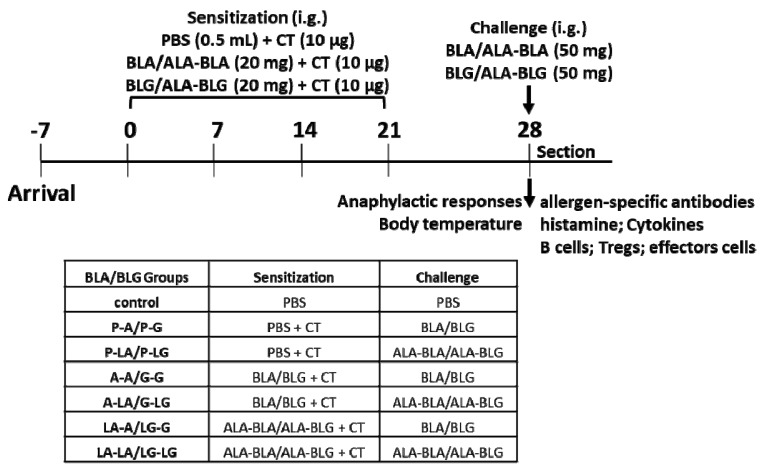
Sensitization and challenge experiments. Treatment scheme: mice were sensitized weekly by i.g. with BLA, ALA–BLA, BLG, or ALA–BLG in PBS using CT as an adjuvant and challenged with these protein preparations. The detailed experimental set-up with *n* = 10 mice per group is shown in the insert: control, sensitized PBS + challenged PBS; P–A, sensitized CT + challenged BLA; P–LA, sensitized CT + challenged ALA–BLA; A–A, sensitized BLA + challenged BLA; A–LA, sensitized BLA + challenged ALA–BLA; LA–A, sensitized ALA–BLA + challenged BLA; LA–LA, sensitized ALA–BLA + challenged ALA–BLA; P–G, sensitized CT + challenged BLG; P–LG, sensitized CT + challenged ALA–BLG; G–G, sensitized BLG + challenged BLG; G–LG, sensitized BLG + challenged ALA–BLG; LG–G, sensitized ALA–BLG + challenged BLG; LG–LG, sensitized ALA–BLG + challenged ALA–BLG. BLA: α-Lactalbumin; BLG: β-lactoglobulin; ALA: α-linolenic acid; CT: cholera toxin; PBS: phosphate buffered saline.

**Figure 2:**
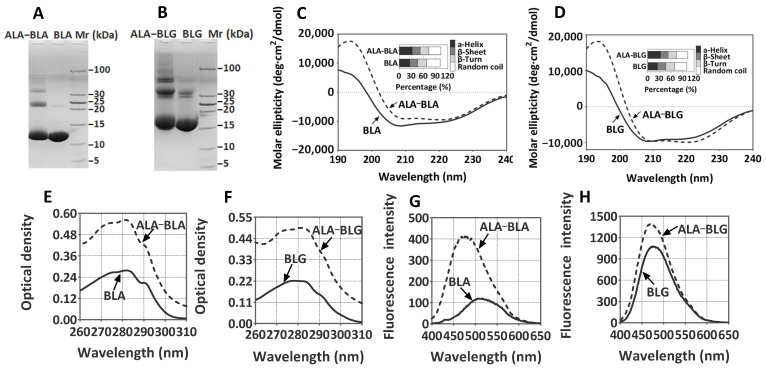
Characterization of ALA–BLA and ALA–BLG: (**A**,**B**) nonreducing tricine–SDS–PAGE of ALA–BLA and ALA–BLG; (**C**,**D**) CD spectra of ALA–BLA and ALA–BLG. The inset shows the secondary structural components of the samples; (**E**,**F**) UV absorption spectra of ALA–BLA and ALA–BLG; (**G**,**H**) ANS fluorescence spectra of ALA–BLA and ALA–BLG.

In this study, we assessed the sensitizing capacity of ALA–BLA/ALA–BLG complexes in comparison with BLA/BLG and also determined the potency of ALA–BLA/ALA–BLG complexes to induce an allergic response (challenge phase) in sensitized mice. The experimental setup resulted in two different treatment groups (BLA and BLG groups, Figure 1). For BLA groups, the mice were randomly divided into seven groups (*n* = 10/group). All mice were sensitized by intragastrically (i.g.) injecting with a blunt needle on days 0, 7, 14, and 21, and oral challenging on day 28. Specifically, the first group was the control group that was sensitized and challenged with 0.5 mL of PBS only. The second (P–A) and third groups (P–LA) were sensitized with 10 μg cholera toxin (CT; Sigma-Aldrich, St. Louis, MO, USA) adjuvant in 0.5 mL of PBS and challenged with 50 mg BLA or ALA–BLA in 0.5 mL of PBS. The fourth (A–A) and fifth groups (A–LA) were sensitized with 20 mg BLA containing 10 μg CT in 0.5 mL of PBS and challenged with 50 mg BLA or ALA–BLA in 0.5 mL of PBS. The sixth (LA–A) and seventh groups (LA–LA) were sensitized with 20 mg ALA–BLA containing 10 μg CT in 0.5 mL of PBS and challenged with 50 mg BLA or ALA–BLA in 0.5 mL of PBS. The BLG groups were also administered in the same way as the BLA groups, as listed in Figure 2A. Then, 40 min after the challenge, the mice were bled by orbital extraction under isoflurane anesthesia, followed by cervical dislocation and section. Anaphylactic symptoms and body temperature were determined as clinical-related symptoms by using a validated anaphylactic scoring (Table S1) and infrared thermometer (Fluke F59, Beijing, China). Blood/serum/plasma, peritoneal cells, spleen, Peyer patch (PP), and mesenteric lymph nodes (MLN) were harvested for further analyses.

### 2.4. Determination of IgE, IgG, IgG1, and IgG2a Antibodies Specific to BLA/BLG and ALA–BLA/ALA–BLG

IgE, IgG, IgG1, and IgG2a antibodies specific to BLA/BLG and ALA–BLA/ALA–BLG were quantified in serum by indirect ELISA. In brief, 96-well plates were precoated with 100 µL of protein at a concentration of 5 μg/mL for BLA or ALA–BLA, or 25 μg/mL for BLG or ALA–BLG, in 50 mM sodium carbonate buffer (pH of 9.6) and then incubated overnight at 4 °C. After washing, the plates were blocked for 1 h at 37 °C with 3% gelatin (Sigma-Aldrich) in PBS, followed by washing three times and serum samples incubation for 1 h (1:5 diluted for IgE, 1:80 or 1:1500 diluted for IgG, 1:100 or 1:1500 diluted for IgG1, and 1:10 or 1:30 diluted for IgG2a). Subsequently, the plates were incubated with horseradish peroxidase (HRP)-labeled anti-mouse IgE, IgG, IgG1, and IgG2a (1:5000, Sigma-Aldrich) for 1 h. Then, a substrate (3,3′,5,5′-tetramethylbenzidine, Neobioscience, Shanghai, China) was added and catalyzed by the HRP to generate the enzymatic signal, and 2 M H_2_SO_4_ was used to stop the reaction. The optical density of each well was determined immediately by using a microplate reader (model 1860, Bio-Rad, Hercules, CA, USA) set to 450 nm. The relative IgG/IgG_1_/IgG_2a_/IgE antibody levels were calculated using the following equation: relative fold change = OD_t_/OD_c_, where OD_t_ and OD_c_ are the absorbance values of the test sample and the control, respectively.

### 2.5. PP, MLN, Spleen, and Peritoneal Cell Processing

PP and MLN samples were aseptically collected and thoroughly disrupted with tissue homogenizer in 5 mL of Dulbecco’s phosphate-buffered saline (DPBS, Sigma-Aldrich) medium. Then, the PP and MLN homogenates were centrifuged at 150× *g* for 10 min, and cell pellets were collected for flow cytometric analysis. To obtain the single-cell suspensions of splenocytes, we ground the spleen samples in 7 mL of 1 × RBC lysis buffer (SorlaBio Co., Beijing, China) and then filtered through nylon mesh. To collect the peritoneal cells, we injected 5 mL of DPBS into the mouse’s peritoneal cavity. Afterward, the abdomen was massaged gently for 1 min and the peritoneal cell-containing fluid was collected, followed by centrifugation at 150× *g* for 10 min to precipitate the peritoneal cell population for flow cytometric analysis.

### 2.6. In Vitro Cytokines, Histamine, and Mouse Mast Cell Protease-1 (mMCP-1) Measurement

Splenocytes were cultured in RPMI-1640 complete medium (Gibco, containing 10% fetal bovine serum, 2 mM L-glutamine, 25 mM HEPES buffer, 100 IU/mL penicillin, and 100 mg/mL streptomycin) at the concentration of 4 × 10^6^ cells per well and stimulated with 40 μg proteins (BLA, LA–BLA, BLG, or LA–BLG) for 72 h (37 °C and 5% CO_2_). Then, the supernatants were frozen at −80 °C until use. All supernatants were thawed simultaneously and assayed to determine the concentrations of secreted cytokines with ProcartaPlex™ Multiplex Immunoassay Kit (Thermo Fisher Scientific, Vienna, Austria), according to the manufacturer’s instructions. These kits allowed the simultaneous quantification of 11 cytokines, including TH1 (IFN-γ, IL-2, and TNF-α), TH2 (IL-4, IL-5, IL-6, and IL-13), TH17 (IL-17A and IL-23), TH9 (IL-9), and Tregs cytokines (IL-10).

The total plasma histamine and serum mMCP-1 levels were measured using commercially available mouse histamine (Demeditec, Berlin, Germany) and mMCP-1 (Neobioscience, Shanghai, China) ELISA kits, in accordance with the manufacturer’s protocol. Individual plasma and serum samples were obtained after challenge and stored at −20 °C before use.

### 2.7. Subpopulation Analysis of the T lymphocytes and Mast Cells by Flow Cytometry

Fluorophore-conjugated antibodies with specificity to the mouse cell surface and intracellular antigens were purchased from eBioscience (San Diego, CA, USA). For surface staining, isolated cells from PP, MLN, and spleen were stained with anti-CD3-APC (145-2C11), anti-CD4-FITC (RM4-5), and anti-CD8a-PE (53-6.7) for T lymphocyte measurements. Peritoneal cells were stained with anti-FcεRI-PE (MAR-1) and anti-CD117-FITC (2B8) for mast cell identification. Lastly, a mouse regulatory T cell staining kit (eBioscience) was used for the Treg cell analysis of splenocytes, PP, and MLN cells, following the manufacturer’s instructions. AccuriTM C6 Plus flow cytometer (BD Biosciences, CA, USA), BD AccuriTM C6 Plus Software, and FlowJo software were used for flow cytometry analysis.

### 2.8. Statistical Analysis

Graphical presentation and statistical analysis of data were performed using the GraphPad Prism software version 7.0 (GraphPad Software Inc., San Diego, CA, USA). All data were expressed as the mean ± SEM of the corresponding parameter. To detect differences between the treated and control groups, one-way or two-way ANOVA, followed by Tukey’s multiple comparisons test for multiple comparisons, was performed to check for significant differences. Different letters indicated statistically significant differences (*p* < 0.05).

## 3. Results

### 3.1. ALA–BLA and ALA–BLG Complex Preparation and Characterization

Previous studies have shown that PUFAs can interact with BLA and BLG to form protein–ligand complexes [18,27]. In this regard, we initially reidentified the ALA–BLA and ALA–BLG complexes and assessed the conformational changes in BLA and BLG after interaction with ALA. As we expected, ALA interaction induced BLA and BLG to form cross-linked components, but no other low-molecular-weight bands were observed (Figure 2A,B). Based on the original bands of BLA (14.2 kDa) and BLG (18.3 kDa), the ALA–BLA and ALA–BLG complexes demonstrated obvious polymer bands, especially those appearing at 24, 42, and 56 kDa, indicating ALA interaction induced BLA and BLG to form aggregates. CD spectra analysis revealed only a slight change in the secondary structures of ALA–BLA and ALA–BLG that were associated with a small increase in α-helix and ß-sheet amounts. However, the differences between ALA–BLA/ALA–BLG and BLA/BLG were insignificant (Figure 2C,D). By contrast, the tertiary structures of ALA–BLA and ALA–BLG were substantially altered based on UV absorption spectra (Figure 2E,F) and ANS fluorescence spectra (Figure 2G,H). Compared with native BLA and BLG, the UV absorbances of ALA–BLA and ALA–BLG increased by 2.02- and 2.23-fold, respectively. Meanwhile, the surface hydrophobicity of LA–BLA and LA–BLG increased by 3.51- and 1.03-fold, respectively.

### 3.2. Sensitizing and Eliciting Potential of ALA–BLA and ALA–BLG Complexes

**Figure 3:**
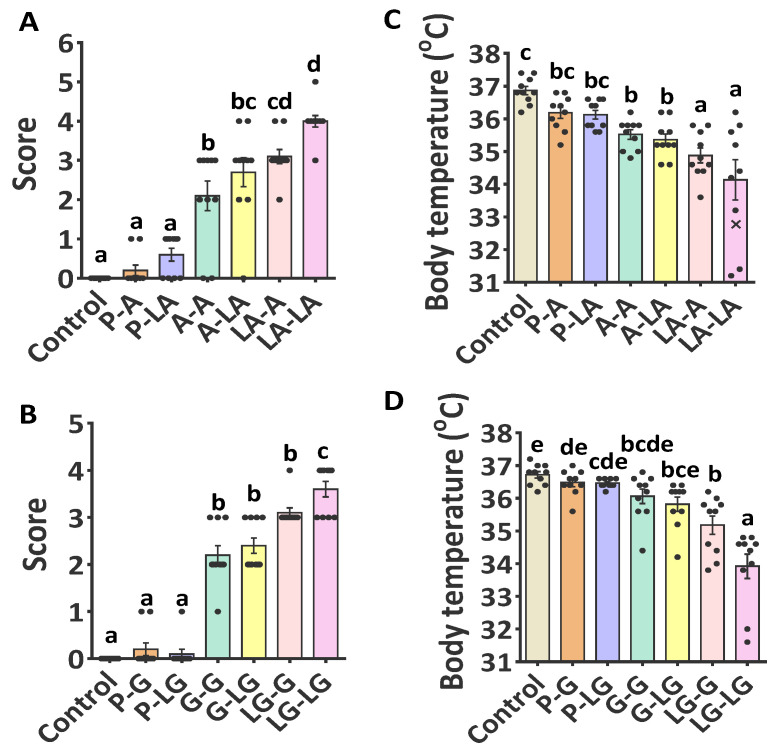
Score of hypersensitivity symptoms (**A**,**B**) and body temperature (**C**,**D**) in BLA and BLG group were followed by i.g. challenges 40 min apart. The symptoms of hypersensitivity were scored on a scale from 0 (no symptoms) to 5 (death), as described in Table S1. Each point represented data from an individual mouse, and values were expressed as means ± SEM (*n* = 10). The results are representative of two independent experiments. Statistical significance was determined by one-way ANOVA, followed by Tukey’s multiple comparisons test. The groups without the same letter in the histogram bar have statistical differences (*p* < 0.05).

Considering that ALA enhanced the alteration of proteins structure prior to interaction with BLA and BLG, we further investigated whether the formation of ALA–BLA/ALA–BLG complexes would change its sensitizing capacity and potency to induce the elicitation phase in sensitized mice compared with BLA/BLG. In the next experiments, we designed sensitization and challenge timelines for the animals, as outlined in Figure 1. The anaphylactic shock symptoms were especially evident in ALA–BLG-sensitized groups, compared with BLG-sensitized groups (Figure 3B). The ALA–BLG-challenged group showed a significant increase in the anaphylactic shock symptoms, compared with the BLG-challenged group, only under the sensitization with ALA–BLG conditions (Figure 3B), thereby indicating that an allergic response was induced in the mice sensitized with ALA–BLG. Simultaneously, compared with BLG, body temperature (Figure 3D) significantly decreased in ALA–BLG in both sensitization and challenge phases. Similar results were obtained in the ALA–BLA complex (Figure 3A,C). The results above showed that ALA–BLA and ALA–BLG complexes have the potential to enhance sensitization and trigger allergic reactions.

### 3.3. Specificity of the Generated Antibodies

**Figure 4:**
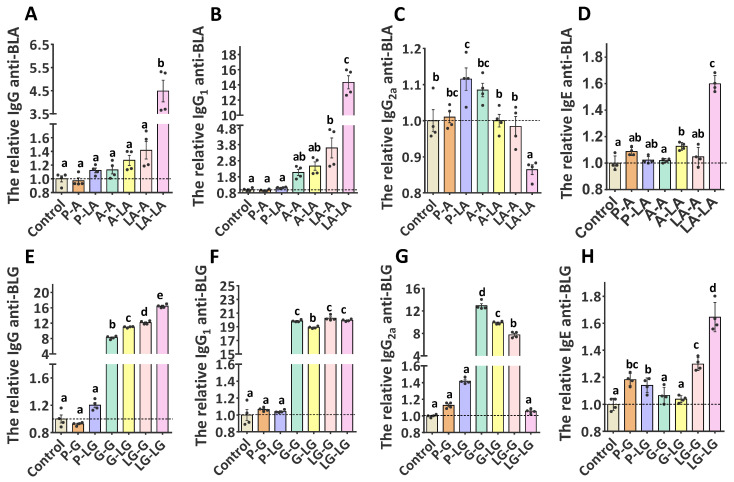
The relative antibodies levels from serum samples of mice determined by direct ELISA: BLA- and BLG-specific IgG (**A**,**E**), IgG_1_ (**B**,**F**), IgG_2a_ (**C**,**G**), and IgE (**D**,**H**) levels were measured in serum of sensitized and challenged mice with BLA, ALA–BLA, BLG, or ALA–BLG, respectively. Each point represented data from an individual mouse, values were expressed as means ± SEM (*n* = 4). The results are representative of two independent experiments. Statistical significance was determined by one-way ANOVA, followed by Tukey’s multiple comparisons test. The groups without the same letter in the histogram bar have statistical differences (*p* < 0.05).

To determine the potential enhancement effects of ALA–BLA and ALA–BLG complexes on humoral immunity, we first evaluated the activation status of B cells (B220^+^ cells). The results demonstrated that the group sensitized and challenged with ALA–BLA/ALA–BLG had significantly higher B220 expression than that of the group sensitized and challenged with BLA/BLG (Figure S2). To verify whether the activation status of B cells can affect humoral adaptive immune responses against BLA/BLG or ALA–BLA/ALA–BLG, we further measured the IgG, IgG1, IgG2a, and IgE levels in the sera. As shown in Figure 4A–D, the group sensitized and challenged with ALA–BLA showed increased BLA-specific IgG/IgG1/IgE levels but reduced IgG2a antibody levels, which significantly differed from those of ALA–BLA-sensitized/BLA-challenged and BLA-sensitized/ALA–BLA-challenged groups. A similar trend for ALA–BLA-specific IgG/IgG1/IgE/IgG2a antibody levels was also observed (Figure S3A–D). Regardless of the sensitization or challenge phase, both BLG and ALA–BLG could elicit an enhanced response, but ALA–BLG significantly increased BLG-/ALA–BLG-specific IgG/IgE levels and reduced IgG2a levels when compared with BLG, and it significantly increased ALA–BLG-specific IgG1 level but not the BLG-specific IgG1 level (Figure 4E–H and Figure S3E–H). These results suggested that conformational B-cell epitopes were affected in the altered ALA–BLA and ALA–BLG structures, which promoted the sensitization and challenge phases.

### 3.4. T-Cell and Cytokine Responses after ALA–BLA and ALA–BLG Complexes Challenge

**Figure 5:**
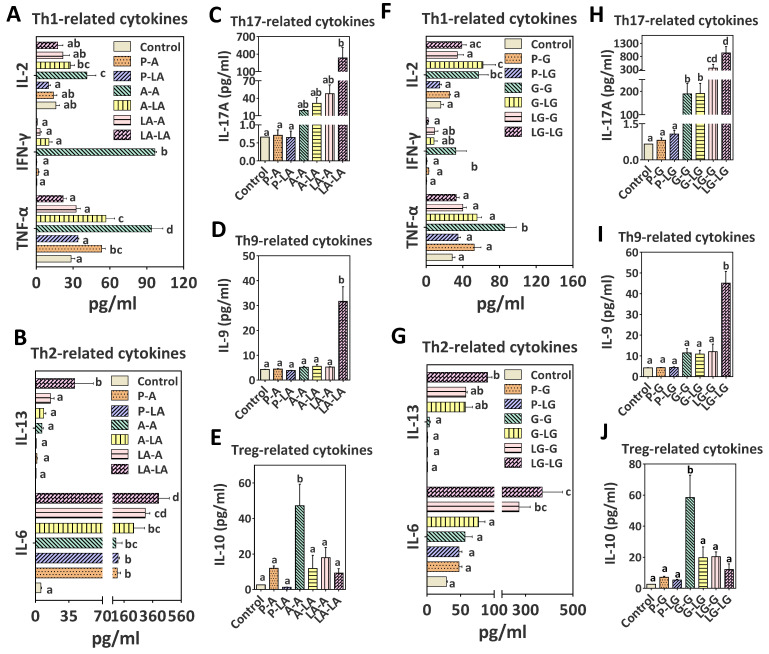
Production of Th1- (**A**,**F**), Th2- (**B**,**G**), Th17- (**C**,**H**), TH9- (**D**,**I**), and Treg-related (**E**,**J**) cytokines in spleen cell cultures. Individual splenocytes (4 × 10^6^) of mice were stimulated with BLA (P–A, A–A, and LA–A groups), ALA–BLA (P–LA, A–LA, and LA–LA groups), BLG (P–G, G–G, and LG–G groups), or ALA–BLG (P–LG, G–LG, and LG–LG group) for 72 h. After 3 days of incubation, cytokines concentrations (pg/mL) in supernatants were measured by using a ProcartaPlex™ Multiplex Immunoassay Kit (Thermo Fisher Scientific, Vienna, Austria). Data were expressed as means ± SEM of six independent mice. The results are representative of two independent experiments. Statistical significance was determined by one-way ANOVA (**C**–**E**,**H**–**J**) and two-way ANOVA (**A**,**B**,**F**,**G**), followed by Tukey’s multiple comparisons test. The groups without the same letter in the histogram bar have statistical differences (*p* < 0.05).

For cellular immune response, TH1- (IFN-γ, IL-2, and TNF-α), TH2- (IL-4, IL-5, IL-6, and IL-13), TH17- (IL-17A and IL-23), TH9- (IL-9), and Tregs-related (IL-10) cytokines were measured in the culture supernatants of splenocytes. First, we identified the percentage of proliferating effector T cells among mouse splenocytes after different proteins stimulations (BLA or ALA–BLA). A tendency toward an increase in CD4^+^ T cells percentages was observed in the group sensitized and challenged with the ALA–BLA, compared with other groups (data not shown). At the T-cell level, we conducted Th1- related cytokines assays to differentiate whether ALA–BLA was responsible for the induction of the protective cytokines IL-2, IFN-γ, and TNF-α by innate immune cells or antigen-specific T cells. As shown in Figure 5A, the group sensitized and challenged with ALA–BLA significantly suppressed IL-2/IFN-γ/TNF-α secretion. However, after antigen-specific stimulation, Th2-related cytokines such as IL-6 and IL-13 significantly increased in different degrees in the group sensitized and challenged with ALA–BLA, compared with other groups (Figure 5B). The difference among the groups in terms of IL-4 and IL-5 secretion levels was insignificant (data not shown). The production of Th17- (IL-17A) and Th9-related (IL-9) cytokines was also enhanced (Figure 5C,D), whereas Tregs-related (IL-10) cytokine (Figure 5E) was reduced in the ALA–BLA group. A similar parent in cytokine profile was observed for Th1/Th2/Th17/Th9/Tregs-related cytokines with ALA–BLG complex (Figure 5F–J).

### 3.5. Identification of Effector Cell Activation

**Figure 6:**
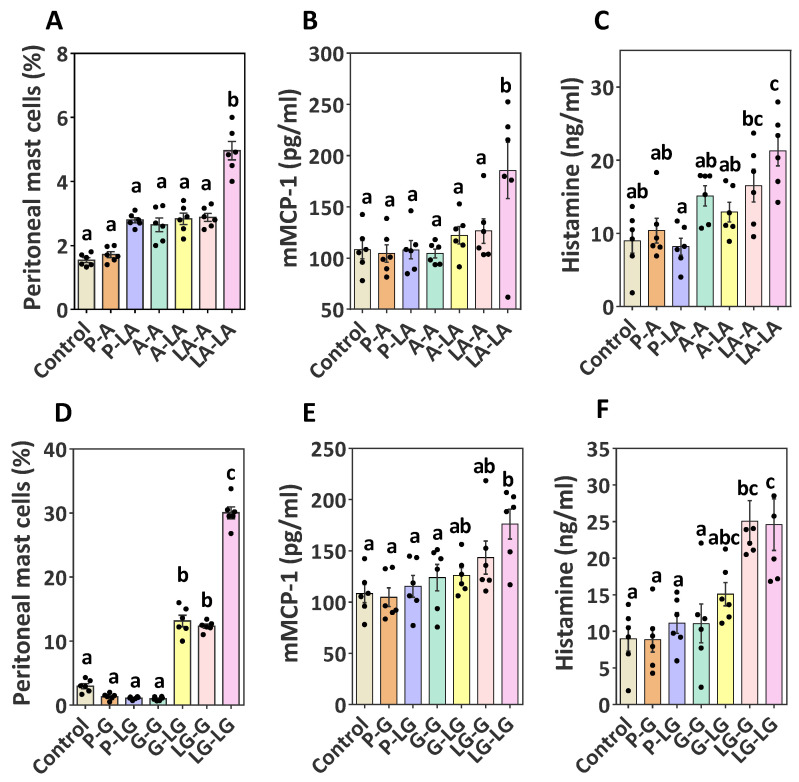
Identification of mast cell activation: (**A**,**D**) percentages of peritoneal FcεRI^+^c-kit^+^ cells in the BLA and BLG groups, respectively; (**B**,**E**) levels of mMCP-1 measured in serum of the BLA and BLG groups, respectively; (**C**,**F**) release of histamine measured in plasma. Data were expressed as means ± SEM of six independent mice. The results are representative of two independent experiments. Statistical significance was determined by one-way ANOVA, followed by Tukey’s multiple comparisons test. The groups without the same letter in the histogram bar have statistical differences (*p* < 0.05).

To explain the allergic response mechanism further, we evaluated the effector cell activation. As primary effector cells, peritoneal mast cells were collected and tested (FcεRI^+^c-kit^+^) by flow cytometry; the gating strategy and representative dot plots of peritoneal FcεRI^+^c-kit^+^ cells are shown in Figure S4A. The percentage of FcεRI^+^c-kit^+^ cells in the group sensitized and challenged with ALA–BLA was much higher than that of the BLA-sensitized groups (Figure 6A). Serum mMCP-1, which is another marker of allergen-induced mast cell activation, was also remarkably enhanced in the group sensitized and challenged with ALA–BLA, compared with that in the other groups (Figure 6B). Similarly, the expression of FcεRI^+^c-kit^+^ mast cells (Figure 6D) and serum mMCP-1 (Figure 6E) was significantly enhanced when sensitized and challenged with ALA–BLG. Finally, we observed increased histamine levels in the group sensitized and challenged with ALA–BLA/ALA–BLG, compared with those of the group sensitized and challenged with BLA/BLG (Figure 6C,F). These findings suggested that ALA–BLA and ALA–BLG enhanced the development of allergic clinical manifestations, which was consistent with the observed hypersensitivity symptoms and body temperature described above (Figure 3).

### 3.6. ALA–BLA and ALA–BLG Complexes Modulated Treg Phenotypes

**Figure 7:**
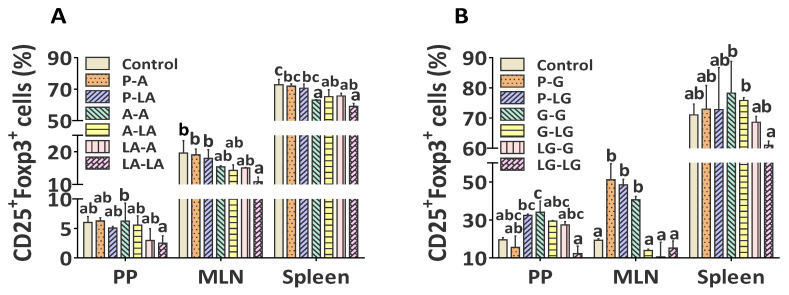
Regulatory T cells (Tregs) identification in spleen, Peyer’s patches (PP), and mesenteric lymph nodes (MLN). Percentages of CD25^+^Foxp3^+^ cells in spleen, PP, and MLN tissues of BLA (**A**) and BLG (**B**) groups. Data were expressed as means ± SEM of four independent mice. The results are representative of two independent experiments. Statistical significance was determined by two-way ANOVA, followed by Tukey’s multiple comparisons test. The groups without the same letter in the histogram bar have statistical differences (*p* < 0.05).

Considering that Tregs play major roles in the regulation of allergic reactions by reducing the magnitude of allergen-activated Th2 responses inducing immune tolerance to allergens [28,29], we further investigated the roles of ALA–BLA and ALA–BLG complexes in modulating Tregs in the mouse PP, MLN, and spleen tissues, and the percentages of CD25^+^Foxp3^+^ cells after gating CD4^+^ cells isolated from spleen tissue are shown in Figure S4B. Regarding the Tregs frequency from mouse PP, MLN, and spleen, the percentage of CD25^+^Foxp3^+^ Tregs within CD4^+^ population in the group sensitized and challenged with ALA–BLA decreased in PP, MLN, and spleen, whereas a significant decrease was observed only in PP and MLN, compared with the group sensitized and challenged with BLA (Figure 7A). Compared with the group sensitized and challenged with BLG, the Tregs percentage in the group sensitized and challenged with ALA–BLG was remarkably decreased in PP, MLN, and spleen (Figure 7B). These results suggested that ALA–BLA and ALA–BLG efficiently inhibited Tregs at the PP, MLN, and spleen, thereby allowing further enhancement in allergic reaction sensitization and elicitation. Finally, we identified that the morphological structure of the intestine and lung was damaged in the groups sensitized and challenged with ALA–BLA/ALA–BLG, compared with those sensitized and challenged with BLA/BLG (Figure S5), which was consistent with the observed allergic clinical manifestations described above.

## 4. Discussion

BLA and BLG are the main target CM proteins for anaphylactic reactions. In the human diet, CM allergenic sensitization is a multifactorial process that is influenced by the allergens (intrinsic factors) as well as by other small molecular compounds (extrinsic factors) in the food matrix, such as fatty acids (FAs), that are directly bound as ligands by the allergen or are present in the allergen source [11,30]. To our knowledge, for more than 20 years, different studies showed that ALA and other C18 PUFAs, as types of lipid components, can combine with BLA and BLG to form complexes in inducing apoptosis in tumor and immature cells but not in healthy differentiated cells [31,32,33]. Among C18 PUFAs, linoleic acid and other ω-6 C18 PUFAs could sufficiently mount a Th2 response and promote the risk of allergy development, but ALA as ω-3 PUFAs possess protective properties for human health by impacting immunological reactions and effectively decreasing the risk of food allergy [34]. Some in vivo and in vitro studies have confirmed that ω-3 PUFAs could protect against the development of food allergy by suppressing Th2 associated cytokine expressions and Fcε receptor I-mediated mast cell activation [35,36]. However, prior to this study, the importance of potential FA interaction capacity in influencing the allergic sensitization and challenge of BLA and BLG in a mouse model remains unclear. Accordingly, exploring the mechanism of the influence of the biological components on the allergenic reactions of BLA and BLG is important. Herein, in this study, we put forward a hypothesis that ALA (a proof-of-concept model based on essential consumption in diet that humans cannot synthesize, which exist in several commercial foods such as dairy products) in diet can interact with BLA/BLG, and the interaction have a potential effect in their allergenic capacity. Interestingly, our results strongly support that the allergic potential of the major milk allergens (BLA and BLG) could be strengthened by the interaction of ALA.

In this study, we first showed the ALA–BLA and ALA–BLG complexes and confirmed the significant alternations of their conformation. Only aggregation was generated when ALA interacted, which was similar to BLA interaction with oleic acid [37], BLG interaction with linoleate [38], and ovalbumin (OVA) interaction with linoleic acid [39]. ALA–BLA and ALA–BLG also promoted protein structure unfolding and increased surface hydrophobicity, which may explain why FAs bind BLA and BLG mainly through hydrophobic interactions [40,41]. Linear and conformational epitopes buried inside the protein are mainly hydrophobic amino acid residues, and the epitopes can be exposed on the protein surface following the gradual unfolding of the structure and the resulting exposure of hydrophobic groups, which could promote the allergenic properties by triggering the intestinal immune responses, and this observation was consistent with previous results [25,42]. Considering the above structural changes in ALA–BLA and ALA–BLG that were closely related to the allergenic properties of BLA and BLG, we further compared the sensitization (induced an increased risk for the development of CMA) and challenge (induced more pronounced allergic symptoms in already allergic mice) phases of ALA–BLA/ALA–BLG with native BLA/BLG in mice. Our results showed indications for a significantly increased risk for the induction of allergic symptoms (anaphylactic shock symptoms and body temperature) when ALA–BLA-/ALA–BLG-sensitized mice were compared with BLA-/BLG-sensitized mice and when BLA-/BLG-sensitized mice were challenged with ALA–BLA/ALA–BLG. These results indicated that ALA induced BLA and BLG to form aggregates that triggered the anaphylactic response. Our findings were similar to a previous report by Roth-Walter et al. [43], demonstrating that the formation of aggregates could enhance allergenic reaction by the disruption of Th1/Th2 balance toward a Th2-polarized immune response. Hence, the next main question on how ALA–BLA and ALA–BLG complexes enhance the humoral (specific antibody production) and cellular immunity (T lymphocyte differentiation and cytokine secretion) in sensitized mice should be answered.

According to the results, ALA–BLA and ALA–BLG upregulated the Th2 response as correlated with IgG, IgG1, and IgE levels and decreased the Th1 response as associated with IgG2a levels. These findings were consistent with previous reports, which showed that lipid components were of paramount importance to induce allergic sensitization by manipulating the shift toward Th2-polarized immune responses [11,44]. For instance, Ber e 1 (2S albumin), which is the major allergen from Brazil nut, required the lipid fraction obtained from the seeds in eliciting IgE responses [45]. Similarly, lipid components were necessary to induce potent allergic capacity of the major mustard (Sin a 2) and peanut (Ara h 1) allergens [30]. It is worth mentioning that the overall enhancement of humoral response in sensitized and challenged mice with ALA–BLA/ALA–BLG was presumably caused by the aid of T lymphocyte, the responsiveness of B cells, or adequate antigen processing.

Next, we investigated the cellular mechanisms involved in ALA–BLA/ALA–BLG-induced allergenic reactions. Multiple studies in animal models indicated that Tregs play important roles in the Th1/Th2 paradigm [28,46,47]. Tregs could induce tolerance to allergens via the generation of inhibitory cytokines such as IL-10 [47,48]. In this study, our results showed that the Treg levels were suppressed in the LA–LA/LG–LG group, and the secretion of IL-10 decreased. Additionally, the LA–LA/LG–LG group demonstrated a significant decrease in Th1-related cytokines (IL-2, IFN-γ, and TNF-β) and an increase in Th2-related cytokines (IL-6 and IL-13) when compared with the A–A/G g group. Those findings suggested that ALA–BLA/ALA–BLG could elevate the sensitization and elicitation capacities by inhibiting Tregs toward a Th2-polarized immune response. These results were similar to those reported by Li et al. [12], demonstrating that medium-chain triglycerides can induce a considerable allergic sensitization and anaphylaxis to OVA in mice, which was associated with a substantial expression of Th2-bias cytokines and an increased uptake through PP. Notably, several reports have shown that Th17 cells, which are associated with IL-17A production, may contribute to the allergic reactions by inhibiting the expression of Th1-related cytokines and consequently modulating the balance between Th1 and Th2 cells [48,49,50], and this conclusion was confirmed by our study. We showed that the IL-17A levels in the sensitized and challenged mice with ALA–BLA/ALA–BLG had a similar increase with Th2 cytokines. Additionally, previous studies also suggested that the allergen-specific production of IL-9 was closely associated with Th2 responses and positively correlated to increased mast cell numbers, thereby indirectly influencing intestinal permeability [51,52]. In agreement with the studies above, ALA interaction with BLA and BLG remarkably increased the IL-9 production ability and mucosal mast cell proliferative capability. A significant increase in mMCP-1 and histamine levels was also detected after ALA–BLA/ALA–BLG challenge, thereby suggesting that mast cell degranulation occurred during the challenge reactions. Our findings provide support for the potential role of IL-9 in CMA pathogenesis, potentially through its influence on intestinal anaphylaxis. Altogether, the above results suggest that ALA interaction will expose most of the BLA/BLG T-cell epitopes, which considerably promoted either the stimulatory or regulatory immune responses. In general, ALA interaction strengthened the allergic potential of the major milk allergens BLA and BLG. When compared with ALA–BLA, ALA–BLG has a stronger potential to promote the sensitization and elicitation of allergic reactions in BALB/c mice. This phenomenon is mainly because the allergenic characteristics of BLG are stronger than those of BLA. BLA, approximately 30–35% of individuals allergic to milk possess specific IgE against BLA. However, regarding BLG, more than 75% of sera from allergic patients possess specific IgE against BLG [53].

In conclusion, our data demonstrated that PUFAs (ALA, as the extrinsic factors of the food matrix) have the potential to promote the sensitization and elicitation of the allergic reaction in BALB/c mice through interaction with BLA and BLG. Furthermore, this study provides a novel understanding of the concept that food matrix plays an important role in food allergy, and allergenicity assessment of proteins should be considered to determine whether there is interaction with the food matrix.

## Data Availability

The authors will send detailed data and calculations on request.

## Supplementary Materials

The following are available online at https://www.mdpi.com/article/10.3390/nu14040822/s1. Table S1 Anaphylactic symptoms scoring table; Figure S1 The analysis of ALA remaining in ALA-treated BLA and BLG complexes. Figure S2 The relative expression of B220 cells from the mouse peripheral blood at 40 min after challenge. Figure S3 The relative antibodies levels from serum samples of mice determined by direct ELISA; Figure S4 Flow cytometric analysis of mast cells and Tregs in BLA and BLG groups. Figure S5 Histological sections (HE) of lung (A-B) and intestine (C-D) tissues from animals challenged by PBS, BLA, LA-BLA, BLG, or LA-BLG, respectively.
