# Supplementary material for: Dietary Linolenic Acid Increases Sensitizing and Eliciting Capacities of Cow’s Milk Whey Proteins in BALB/c Mice
Source: Nutrients. 2022 Feb 16;14(4):822. doi: 10.3390/nu14040822 (PMC8877816; doi:10.3390/nu14040822)
Supplement: Supplementary file 1 [file nutrients-14-00822-s001.zip › nutrients-1576482-supplementary.pdf]

Supplementary Data

Table S1. The scoring table of anaphylactic symptoms.

Score	Symptoms
0	No symptoms
1	Scratching nose and mouth
2	Swelling around the eyes and mouth; reduced activity; high breathing rate
3	Shortness of breath; blue rash around the mouth and tail; higher breathing rate
4	No activity after stimulation; shivering and muscle contractions
5	Death by shock

Figures and Figure Legends

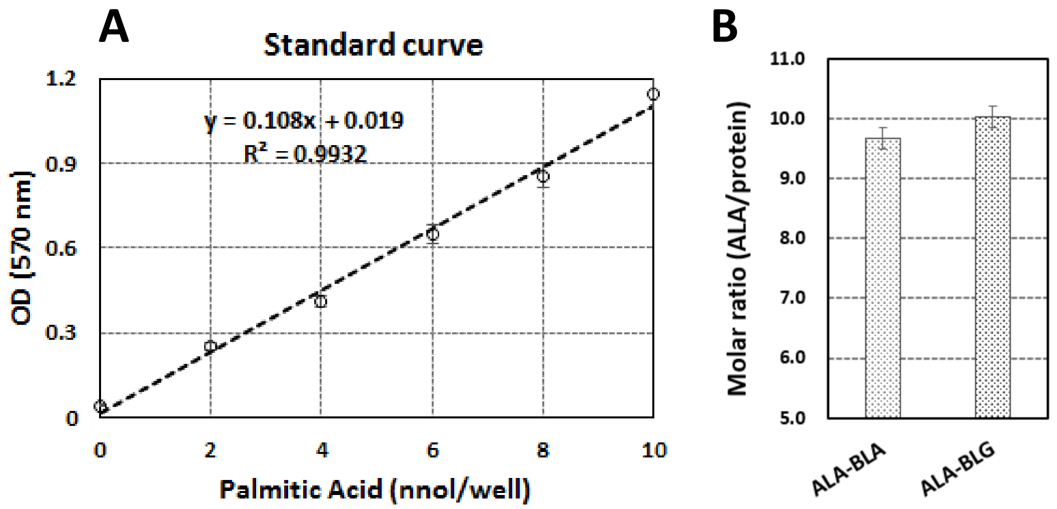

FIGURE S1. (**A**) The palmitic acid standard curve according to the Free Fatty Acid Quantification Kit protocol. Briefly, the samples were added to a 96-well plate in several dilutions. In parallel, a standard curve was prepared with known amounts of palmitic acid. FA assay buffer was added to each well to reach a final volume of 50 μL. Acyl–CoA synthetase (2 μL) was then added to each well to convert FA to their CoA derivatives, followed by incubation at 37°C for 30 min. A solution comprising enzymes, an enhancer, and a FA probe was added to each well to generate the color. After 30 min of incubation at 37°C in the dark, the absorbance was detected at 570 nm using an ELISA plate reader (Model 1860, Bio-Rad, USA). (B) The quantity of ALA remaining in ALA–BLA and ALA–BLG complexes after dialysis. Results are represented as means ± SEM (n=2).

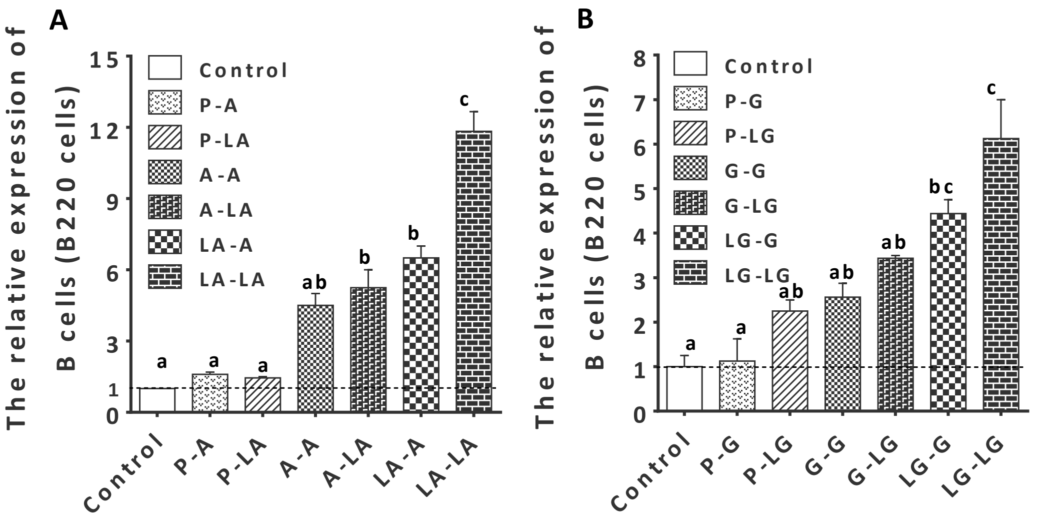

FIGURE S2. The relative expression of B220 cells from the mouse peripheral blood at 40 min after challenge. Values are expressed as means ± SEM (n=2). Different letters indicate statistically significant differences.

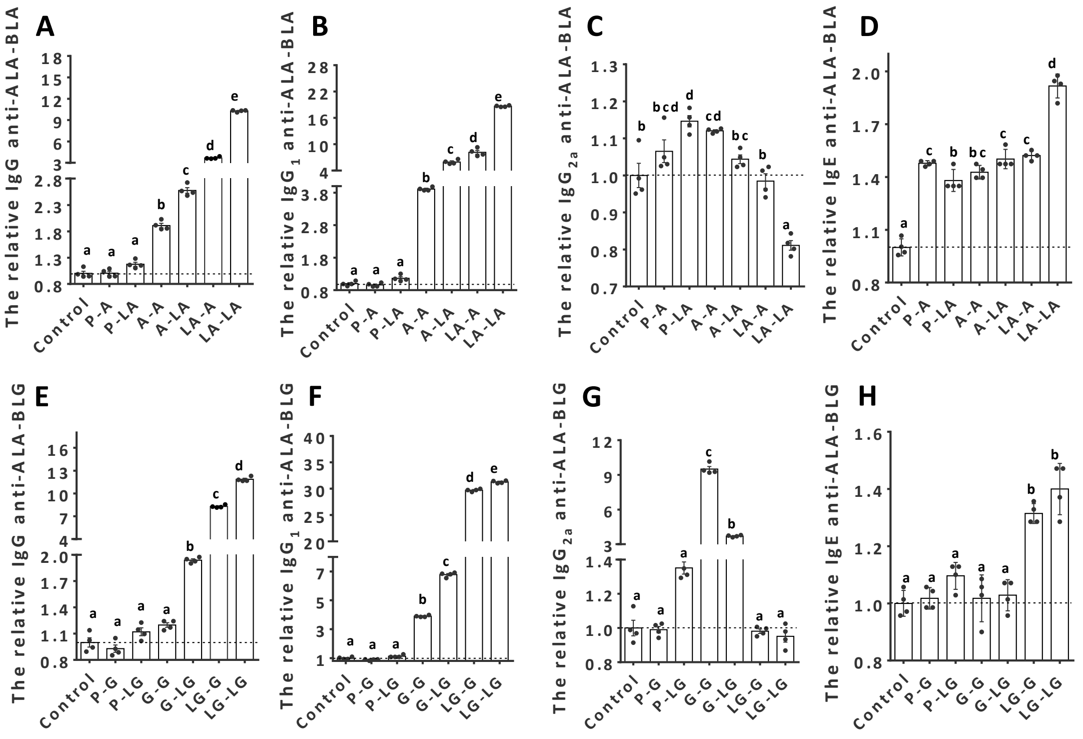

FIGURE S3. The relative antibodies levels from serum samples of mice determined by direct ELISA: ALA–BLA- and ALA–BLG-specific IgG (**A,E**), IgG$_1$ (**B,F**), IgG$_{2a}$ (**C,G**), and IgE (**D,H**) levels were measured in serum of sensitized and challenged mice with BLA, ALA–BLA, BLG, or ALA–BLG, respectively. Each point represented data from an individual mouse, and values are expressed as means ± SEM (n=4). Different letters indicate statistically significant differences.

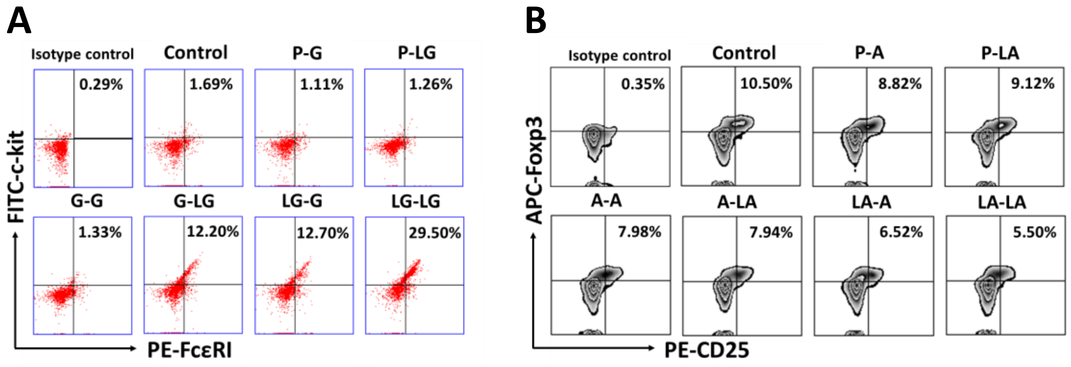

Figure S4. (**A**) Representative dot plots showing percentages of peritoneal FcεRI+c-kit+ cells in the BLG groups; (**B**) representative percentages of CD25+Foxp3+ cells after gating CD4+ cells isolated from the spleen tissue of BLA groups.

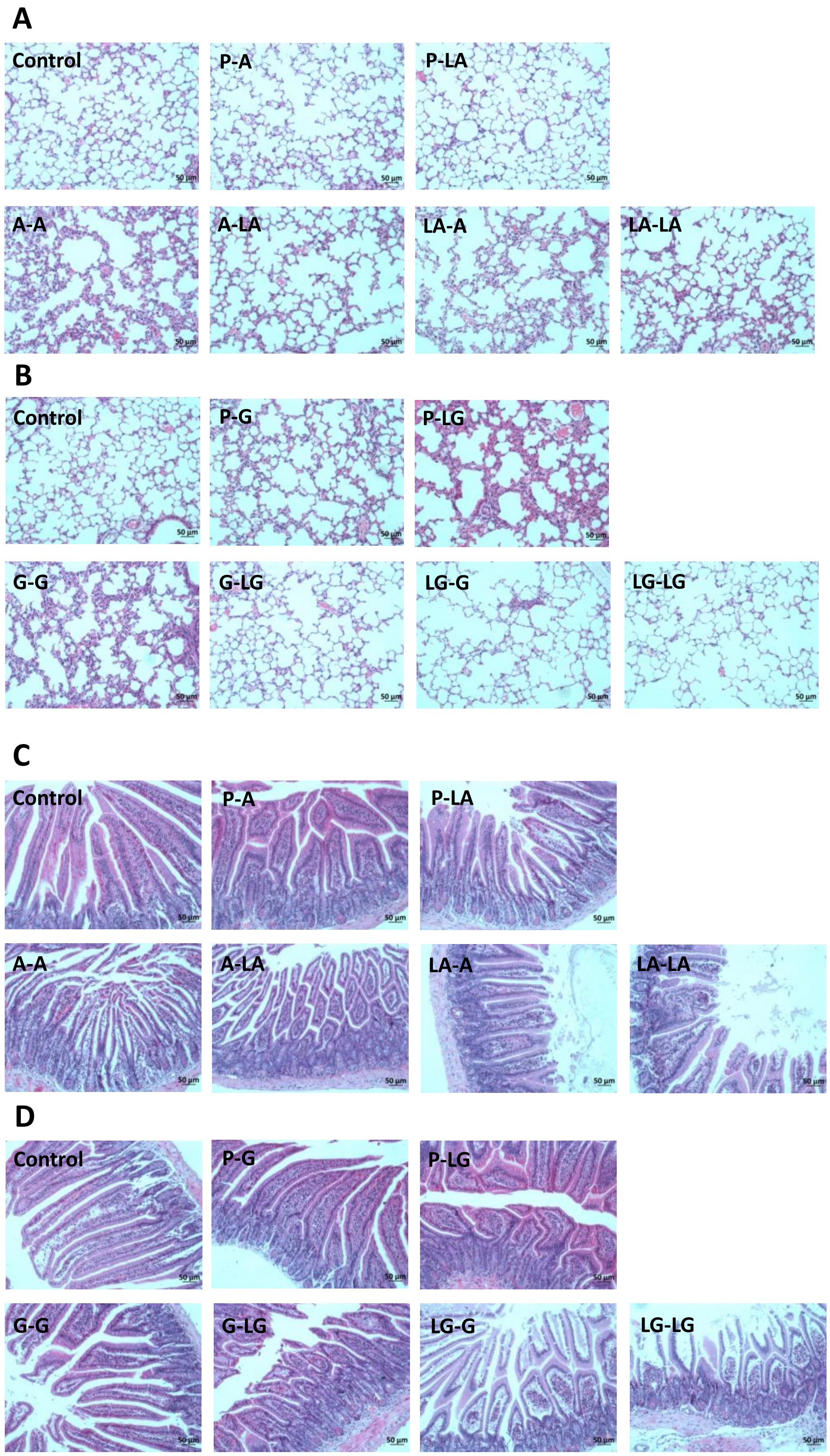

FIGURE S5. Histological sections (HE) of lung (**A,B**) and intestine (**C,D**) tissues from animals challenged by PBS, BLA, ALA–BLA, BLG, or ALA–BLG, respectively.
